# Insertional mutagenesis in *ChordinA* induced by endogenous *ΔTgf2* transposon leads to bifurcation of axial skeletal systems in grass goldfish

**DOI:** 10.1038/s41598-019-40651-1

**Published:** 2019-03-11

**Authors:** Dan-Dan Guo, Yi-Wen Sun, Wen-Tao Cui, Hong-Hong Guo, Shang-Ke Du, Jie Chen, Shu-Ming Zou

**Affiliations:** Genetics and Breeding Center for Blunt Snout Bream, Ministry of Agriculture, Key Laboratory of Freshwater Aquatic Genetic Resources, Ministry of Agriculture, National Demonstration center for Experimental Fisheries Science Education (Shanghai Ocean University), Shanghai, 201306 China

## Abstract

The grass goldfish appeared early in the evolutionary history of goldfish, and shows heritable stability in the development of the caudal fin. The twin-tail phenotype is extremely rare, however, some twin-tail individuals were produced in the process of breeding for ornamental value. From mutations in the twin-tail goldfish genome, we identified two kinds of *Tgf2* transposons. One type was completely sequenced *Tgf2* and the other type was *ΔTgf2*, which had 858 bp missing. We speculate that the bifurcation of the axial skeletal system in goldfish may be caused by an endogenous *ΔTgf2* insertion mutation in *Chordin A*, as *ΔTgf2* has no transposition activity and blocks the expression of *Chordin A*. The twin-tail showed doubled caudal fin and accumulation of red blood cells in the tail. In addition, *in situ* hybridization revealed that ventral embryonic tissue markers (*eve1*, *sizzled*, and *bmp4*) were more widely and strongly expressed in the twin-tail than in the wild-type embryos during the gastrula stage, and *bmp4* showed bifurcated expression patterns in the posterior region of the twin-tail embryos. These results provide new insights into the artificial breeding of genetically stable twin-tail grass goldfish families.

## Introduction

Goldfish (*Carassius auratus*) are one of the three famous ornamental fish, and are known as the “water fairy.” Goldfish evolved from wild crucian carp and mutated into different species (grass goldfish, wen goldfish, dragon-eye goldfish, and oval goldfish). Each species has unique characteristics, but the most characteristic phenotype is the bifurcated caudal fin^1^. The caudal fin of aquatic animals is mainly divided into two types (wild-type and twin-tail) and goldfish are representative species of the twin-tail. The goldfish tail is supported by the caudal axial skeleton, but the twin-tail has bifurcated fin folds that differ from the wild-type. There are many factors that caused bifurcation of the goldfish caudal fin, such as growth environment, genetic factors, and epigenetic effects. Previous reports showed that the development of the goldfish caudal fin is closely related with regulatory factors in the ooplasm^2,3^. In addition, a single nucleotide mutation at the 127^th^ amino acid base in *ChordinA* restricted its transcription regulation, which generated the twin-tail phenotype^4^.

*Tgf2*, the second transposon in vertebrates, belongs to the hAT family and is also found in goldfish. *Tgf2* plays an important role in transgenesis and gene trapping with independent transposition activity^5,6^. Previous research on transgenesis was carried out by coinjecting the vector plasmid and transposase mRNA into zebrafish, grass carp, and goldfish. The results showed that the eGFP reporter gene was transferred from the vector plasmid into grass carp and goldfish genomes, with an incorporation efficiency of 96% and 37%, respectively. In addition, the *Tgf2* transposon in goldfish has three other copies that belong to the goldfish *Tgf2*-Ds group (*ΔTgf2*) and have no independent transposition capability^5,7,8^.

In this study, we obtained the twin-tail phenotype induced by the endogenous *ΔTgf2* transposon insertional mutation and conducted pedigree breeding to obtain a genetically stable twin-tail goldfish strain. We also conducted functional assays and analyzed the expression patterns in ventral embryonic tissue markers.

## Results

### The discrimination of wild-type and twin-tail goldfish

**Figure 1:**
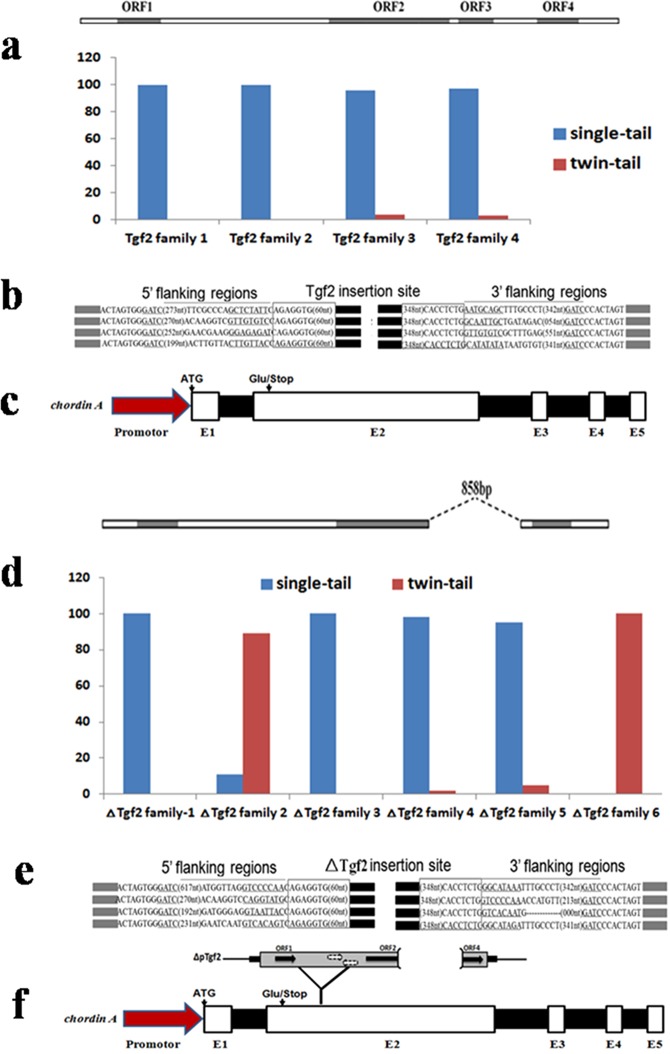
Test of the insertion site of the *ΔTgf2* transposon in grass goldfish. Different families with or without the *Tgf2* transposon were established to analyze the frequency of wild-type and twin-tail grass goldfish, respectively. (**a**,**d**) Observed *Tgf2* and *ΔTgf2* transposon insertion copies (**b**,**e**), and the position of *ChordinA* insertion (**c**,**f**).

In the 10 families (*Tgf2* family-1–4 and *ΔTgf2* family-1–6), all the goldfish from the *Tgf2* family-1 and -2 were wild-type, more than 95% of goldfish from family-3 and -4 were wild-type, and only a few individuals were twin-tail (Fig. 1a). Similarly, all the goldfish from *ΔTgf2* family-1 and -3 were wild-type, more than 92% of goldfish from family-4 and -5 were wild-type, and only a few were twin-tail (Fig. 1d). In contrast, 89% and 100% goldfish from *ΔTgf2* family-2 and -6 were twin-tailed, respectively. As such, we inferred that endogenous *ΔTgf2* may participate in the generation of, but not necessarily induce, the twin-tail phenotype.

To examine how the endogenous *ΔTgf2* contributed to the twin-tail phenotype, we amplified and sequenced cDNA from wild-type and twin-tail goldfish. In the twin-tail goldfish, *ΔTgf2* was found inserted into 401 nt in the 2^nd^ exon of *ChordinA*. We named the gene as *ChordinA*^*ΔTgf2*^ and described the homozygous genotype as *ChordinA*^*ΔTgf2/ΔTgf2*^ (Fig. 1f). Conversely, in the wild-type goldfish, no insert was found in *ChordinA* and we named the gene *ChordinA*^+^, and described the homozygous genotype as *ChordinA*^+/+^ (Fig. 1c). A previous report showed that *ChordinA* plays an important role in the differentiation of the caudal fin in goldfish^4^, and we speculate that the insertion of endogenous *ΔTgf2* leads to mutagenesis in *ChordinA*, which results in bifurcation of the caudal fin.

### Breeding of twin-tail goldfish strains based on the *ΔTgf2* transposon

**Figure 2:**
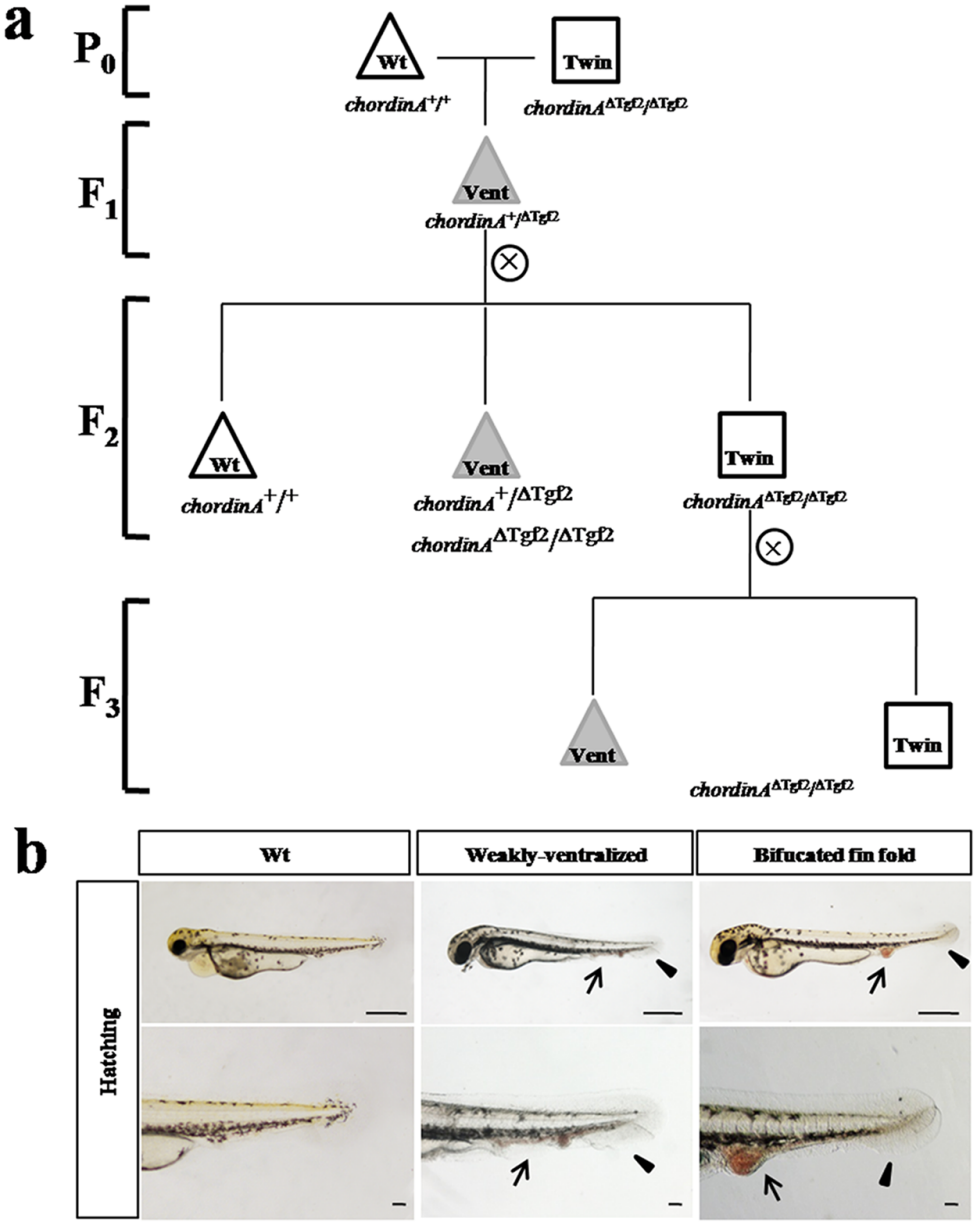
Family breeding design and embryonic observations of the twin-tail grass goldfish. The breeding strain of the twin-tail grass goldfish was obtained using hybridization and selfing. (**a**) Triangles indicate the wild-type phenotype. Square indicates the twin-tail phenotype. Gray, filled triangles indicate the variation in wild-type. In the F2 generation, three phenotypes were observed. (**b**) Arrows point to the red blood cell aggregation and bifurcated caudal fin.

When *ChordinA*^+/+^ individuals were crossed with *ChordinA*^*ΔTgf2/ΔTgf2*^, the F1 group (~375 individuals) were vent group(*ChordinA*^+*/ΔTgf2*^), and the F2 group (~846 individuals) was separated into three types: wild-type (wt) larvae from wild-type (*chordin*^*A*+/+^) embryos (~589 individuals), single caudal fin (vent) larvae from weakly-ventralized (*chordinA*^+*/ΔTgf2*^) or bifurcated caudal fin fold (*chordinA*^*ΔTgf2/ΔTgf2*^) embryos (~21 individuals), and bifurcated caudal fin (twin) larvae from bifurcated fin fold (*chordinA*^*ΔTgf2/ΔTgf2*^) embryos (~236 individuals) (Fig. 2a, Table 1). At 2 dpf, the wt phenotype showed the single tail phenotype. The vent phenotype with the incomplete forked fin folds was characterized by a single tail and the twin phenotype with completely divided fin folds showed twin-tail phenotype. However, the vent and twin individuals have a common characteristic that they have the accumulation of red blood cells in the tail (Fig. 2b). The F3 group, which included 317 twin individuals (93%) and 23 vent individuals (7%), suggested that the vent phenotype may be atavism due to the external environment and/or other genetic factors^9,10^.

**Table 1:** Phenotypes and genotypes of goldfish strains.

Group	Genotype	Phenotype		
		Wt	Vent	Twin
F1	*chordinA* ^+^ */* ^*ΔTgf2*^	375	0	0
F2	*chordinA* ^+/+^	589	21	236
	*chordinA* ^+^ */* ^*ΔTgf2*^			
	*chordinA* ^*ΔTgf2*^ */* ^*ΔTgf2*^			
F3	*chordinA* ^*ΔTgf2*^ */* ^*ΔTgf2*^	0	23	317

### Comparison between wild-type and twin-tail goldfish

**Figure 3:**
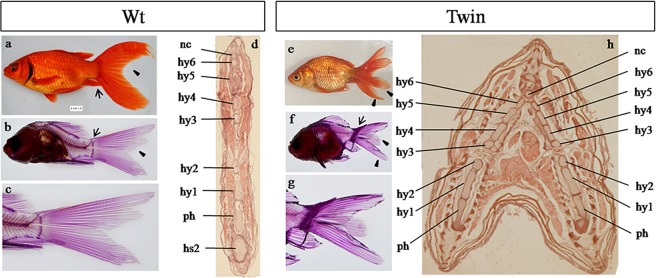
Tail morphology of grass goldfish. Different observation methods were used to obtain the external morphology (**a**,**e**), the skeletal structure (**b**,**f**,**c**,**g**), and the transverse section of the caudal fin. (**d**,**h**) nc (notochord), hy (hypural bone), ph (paranasal bone of the hypural bone), hs (haemal spine).

**Figure 4:**
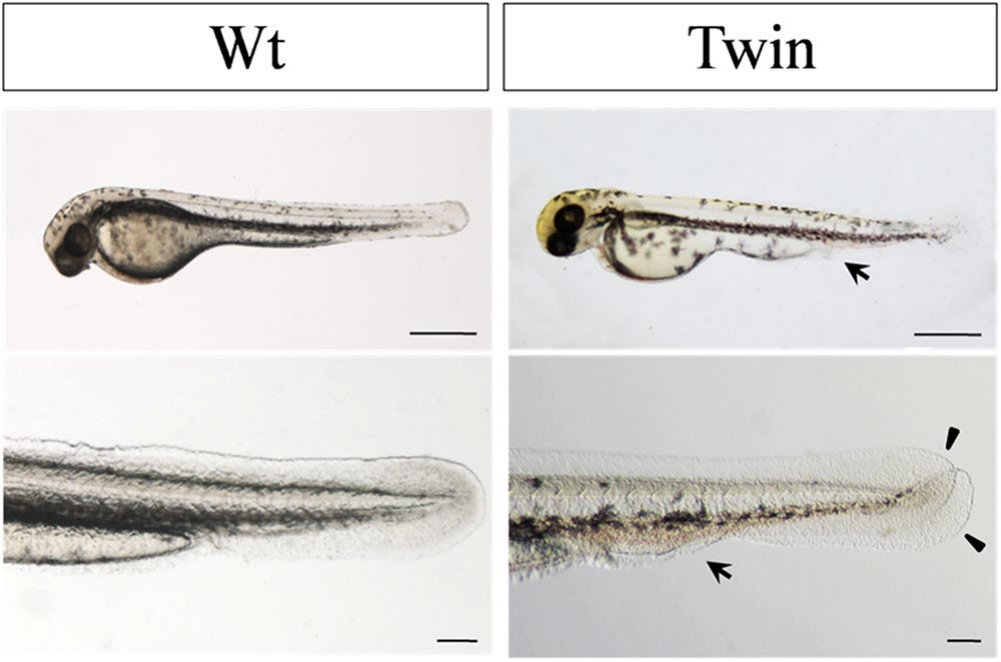
Embryonic observations during the incubation period of the wild-type and twin-tail grass goldfish. Arrows point to the abdominal stem where a large number of red blood cells accumulated in twin-tail grass goldfish.

We compared embryonic and adult fish to distinguish between wild-type and twin-tail goldfish. We observed several different characteristics between the two strains of goldfish. Morphologically, wild-type goldfish share characteristics with wild-type crucian carp (*Carassius auratus*) and have a traditional dovetail-shape tail lobe (Fig. 3a). Meanwhile, in the evolutionary history of the grass goldfish, a twin-tail phenotype appeared with bifurcated fin folds^11^. Alizarin red staining of axial skeletons is a practical method to analyze the anatomical structure of goldfish tails^12–14^. Caudal axial skeletal elements were identified according to previous reports^15^. The results showed that the tail nerve bone of both wild-type and twin-tail goldfish were completely mineralized and formed the caudal fin. The caudal fin were attached to the Ep (epural bone), Hy (hypural bone), and Ph (paranasal bone of the hypural bone), however, the quantification data of Nc (notochord), Ph, and Hy1~6 in the wild-type and twin-tail goldfish showed no obvious difference (Table 2). The number of caudal fin rays in wild-type goldfish was 20 to 24, arranged in a double-leaf type shape (Fig. 3b,c), while that of twin-tail goldfish was 39 to 47, arranged in a four-leaf type shape (Fig. 3f,g). Histologically, the characteristics of the axial skeleton demonstrated that the origin of the twin-tail phenotype was attributable to double germination of Hy and Ph (Fig. 3h), which were developed non-redundantly in the wild-type goldfish (Fig. 3d). Embryological results showed that accumulation of red blood cells in bifurcated fin folds only occurred in twin-tail goldfish, and not in wild-type goldfish (Figs 2b and 4). This was consistent with previous reports^4^.

**Table 2:** The quantification data of nc, ph, and hy1~6 in the Wt and Twin.

Group	Wt		Twin	
Characteristic	Width (cm)	Length (cm)	Width (cm)	Length (cm)
nc	0.037	0.028	0.058	0.058
hy6	0.037	0.05	0.026	0.073
hy5	0.091	0.15	0.039	0.11
hy4	0.05	0.106	0.036	0.125
hy3	0.031	0.072	0.054	0.048
hy2	0.037	0.059	0.054	0.075
hy1	0.05	0.209	0.048	0.221
ph	0.066	0.269	0.048	0.188

### Expression patterns of *Tgf2* and *ΔTgf2* transposon

**Figure 5:**
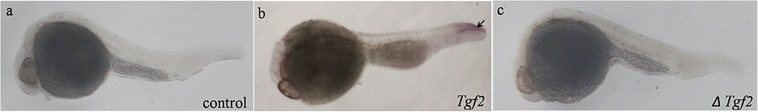
Expression patterns of *Tgf2* and *ΔTgf2* in grass goldfish embryos. Arrows point to the signals.

In order to detect the effect of *Tgf2* and *ΔTgf2* on grass goldfish, we performed *in situ* hybridization of *Tgf2* in wide-type (single-tail) and *ΔTgf2* embryos (twin-tail). The result showed that the expression patterns of *Tgf2* and *ΔTgf2* were different. *Tgf2* transposase mRNA was detected in tail cells in the wide-type embryos (Fig. 5b). However, *Tgf2* transposase mRNA was not detected in *ΔTgf2* embryos (Fig. 5c).

### Dorsal-Ventral (DV) axis development of twin-tail goldfish embryos

**Figure 6:**
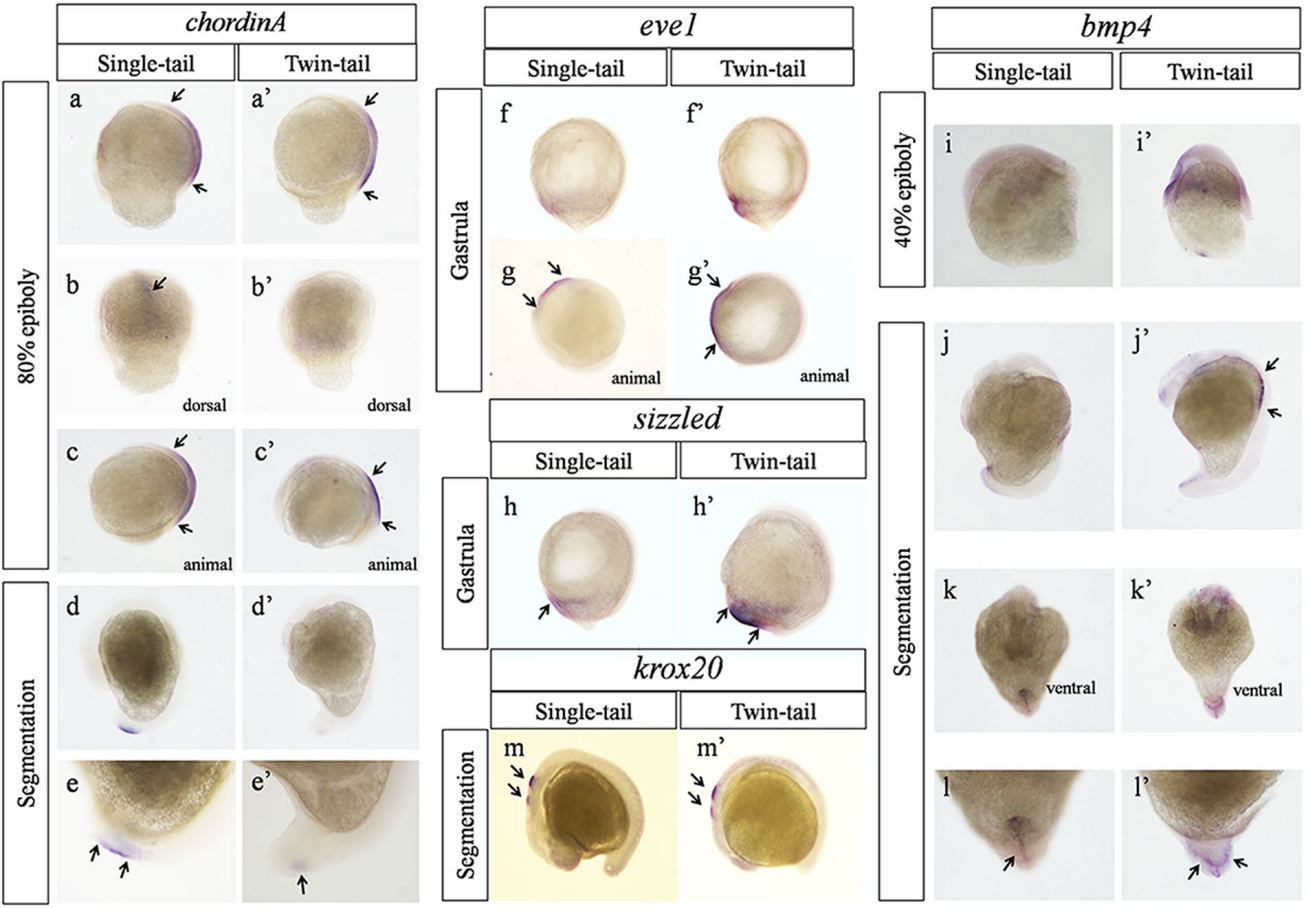
Comparison of the dorsal-ventral (DV) axis gene expression patterns between the wild-type and twin-tail grass goldfish. Expression patterns of *ChordinA* (**a**–e’), *eve1* (**f**,g’), *sizzled* (**h**,h’), *krox20* (**m**,m’), and *bmp4* (**i**–l’).

The previous research showed that dino regulating dorsal development in the zebrafish, the tail is enlarged in dino mutant embryos. The zebrafish mutant ogon (also called short tail) displays ventralized phenotypes similar to the dino mutant. In these mutants, the ventral embryonic tissue markers (eve1, sizzled) are expressed in ventrolateral marginal cells showed different expression patterns. *Krox20* is a marker gene, mainly expressed in the hindbrain, which is usually used as a positive control for other ventral marker genes in the DV axis. Bmps also function in skeletal development and the dorsal-ventral axis of vertebrate embryos is thought to be specified by a gradient of bone morphogenetic protein (bmp) activity^16–18^. To determine how the insertional mutation in *ChordinA* that was mediated by endogenous *ΔTgf2* contributed to DV patterning in twin-tail goldfish embryos, we examined the expression patterns of *ChordinA*, ventral embryonic tissue markers (*eve1*, *sizzled*, and *bmp4*), and a hindbrain marker (*krox20*) in the embryos of wild-type and twin-tail goldfish (F3). The results showed that the expression patterns were different between wild-type and twin-tail embryos. During the gastrula stage (80% epiboly), *ChordinA* was expressed in the outward germ layer and showed wider expression patterns in wild-type (Fig. 6a–c) than in twin-tail embryos (Fig. 6a’–c’). Similarly, during the segmentation stage, *ChordinA* was mainly expressed in the tailbud and showed obviously wider expression patterns in wild-type (Fig. 6d,e) than in twin-tail embryos (Fig. 6d’,e’). A few reports have suggested that *ChordinA* plays an important role in the development of the DV axis and can control the caudal fin phenotype of goldfish^19–22^. On the contrary, during the gastrula stage, *eve1* and *sizzled* showed stronger signal and wider expression patterns in twin-tail (Fig. 6f’–h’) than in wild-type embryos (Fig. 6f–h) in the posterior-ventral regions. *Bmp4* is one of the important determining factors for ventral embryonic development^23,24^. During the late gastrula stage (40% epiboly), the expression of *bmp4* (Fig. 6i’) was stronger in twin-tail than in wild-type embryos (Fig. 6i). In addition, *bmp4* expression patterns in wild-type and twin-tail embryos showed obvious differences during the segmentation stage. As shown in Fig. 6k’,l’, *bmp4* showed bifurcated expression patterns in the posterior region of twin-tail embryos, which were not observed in the wild-type embryos (Fig. 6k,l). This indicated that the axial skeleton became bifurcated and doubled during the segmentation stage in the twin-tail goldfish. The ventral embryonic tissue marker expression patterns were consistent with a previous study of dino zebrafish mutants and other *chordin*-deficient embryos^16,17,25–29^. The results showed that *krox20* expression patterns were not different between wild-type and twin-tail embryos^4^, which differed from previous reports of significantly reduced in *krox20* expression patterns in *chordin*-deficient vertebrate embryos^17,25,27,29,30^. This indicated that the formation of the DV axis played an important regulatory role in the differentiation of tail-patterns in goldfish.

## Discussion

The generation of diversified caudal fins in goldfish is caused by a mutation. Previous studies have found that *Chordin* mutants generate bifurcated caudal axial skeletons^2–4,31^. This natural mutation has been produced and inherited consistently through artificial selection since the song dynasty of China^32^.

The *Tgf2* transposon has not been observed in wild crucian carp, but occurs in grass goldfish as well as other goldfish strains. In the grass goldfish genome, the complete sequence type (*Tgf2*) and the multiple missing pieces type (*ΔTgf2*) have been detected, which indirectly indicated that grass goldfish is the ancestor of many other strains^7^. The *Tgf2* transposon relies on its encoded transposase to mediate translocation. Under the artificial selection, those goldfish whose morphological variation caused by transposon were preserved, this may be because the *Tgf2* transposon retained their independent transposition activity through long-term evolution^33^. Generally, there are large differences between goldfish parents and progeny in terms of color and proportions, which could be due to the insertion mutation of the *Tgf2* transposon^5,33^. However, the *ΔTgf2* transposon are unable to produce transposase and has no independent transposition capability. According to Mendel’s law, *ΔTgf2* homozygote will be generated when the goldfish with *ΔTgf2* was selfed. Therefore, the phenotype caused by the *ΔTgf2* insertion mutation might be genetically stable^5,7,8^. A limited number of individuals with the mutation might have survived. Therefore, we speculated that such a large number of twin-tail goldfish from only 10 families were not directly produced by the insertion mutation, but from several generations of hybridization of insertion mutation individuals. In previous studies, *in situ* hybridization of *Tgf2* showed that it has different expression patterns in different embryos and was detected in the head and tail^6^. In our study, *Tgf2* can be detected in the tail. Our result is consistent with previous reports of *Tgf2* expression patterns^34^. This may be caused by the *Tgf2* has independent transposition capability. The insertion site in the goldfish genome affects the expression of *Tgf2* transposase. However, no transposase mRNA was detected in *ΔTgf2* embryos confirming the *ΔTgf2* with large deletion lost its independent transposition capability. In this study, *ΔTgf2* transposon was found inserted in the 2^nd^ exon of *ChordinA* in the twin-tail goldfish. And it is precisely because *ΔTgf2* has no independent transposition capability, insertional mutagenesis in *ChordinA* inhibited the expression of the 2^nd^ to 4^th^ exons of *ChordinA*. Previous studies showed that a single nucleotide mutation in the 127^th^ amino acid of C*hordinA* (on the 2^nd^ exon, prior to the *ΔTgf2* insertion site) generated a stop codon and produced the twin-tail phenotype, we speculate that the bifurcation of the axial skeletal system in goldfish may be caused by an endogenous *ΔTgf2* insertion mutation in *ChordinA*. However, the relation between the insertional mutation mediated by the *ΔTgf2* transposon and a single nucleotide mutation in *ChordinA* require further study.

Alizarin red staining showed that the tail nerve bones forming the caudal fin of both wild-type and twin-tail goldfish were completely mineralized. The difference was that the elements of the axial skeleton system of twin-tail goldfish were doubled with bifurcated fin folds. *In situ* hybridization indicated that the expression patterns of the two goldfish strains were different during the gastrula stage. The expression of ventral embryonic tissue genes increased significantly due to the *ΔTgf2* insertional mutation in *ChordinA*. This indicated that the ventral tissue had proliferated, which generated the double caudal fin^35^. In addition, *ChordinA* showed wider expression patterns in wild-type goldfish than in twin-tail goldfish embryos, during both the gastrula and segmentation stages. However, the reduced expression of *ChordinA* can also lead to the generation of the twin-tail phenotype. Therefore, we speculate that the insertional mutation in *ChordinA* mediated by *ΔTgf2* contributed to the generation of twin-tail by suppressing the inhibitory effect of *ChordinA*.

Insertional mutagenesis induced by the *ΔTgf2* transposon has mainly been detected in goldfish, so we speculate that this insertional mutation has a special regulatory role in the generation of twin-tail goldfish^5,7,36^. Based on this study, we infer that insertional mutagenesis mediated by the *ΔTgf2* transposon was the main cause of twin-tail; however, it was also affected by other genetic and/or environment factors. By exploring the mechanism of *ΔTgf2* insertional mutagenesis and the generation of the goldfish caudal fin, these results provide a theoretical basis for artificial breeding of genetically stable twin-tail grass goldfish families.

## Materials and Methods

### Experimental goldfish and embryos

P0 generation of wild-type and twin-tail goldfish were obtained from the Genetics and Breeding Center of Shanghai Ocean University, Shanghai, China. Embryos were generated by artificial insemination. Fertilized eggs (~150) were placed in petri dishes (10 cm in diameter) and development at room temperature (22 ± 1 °C). Petri dish with well-aerated water to maintain normal dissolved oxygen (DO) levels during embryogenesis and replaced every 4 hours. Embryos used for RNA extraction were stored by immersion in RNA Store (ABigen, Beijing, China) and kept in 4 °C overnight and then −80 °C until used. Embryos collected for *in situ* hybridization analysis were kept in embryonic medium supplemented with 0.003% (w/v) 2-phenylthiourea to prevent pigmentation, then fixed overnight at 4 °C in 4% paraformaldehyde (PFA) and then −20 °C in methyl alcohol. Juvenile goldfish used for anatomical and histological analyses were sacrificed by immersion in MS-222 (tricaine methanesulfonate, Sigma, St. Louis, MO) for 5 min. All experiments were performed in accordance with the Guide for the Care and Use of Laboratory Animals of the National Advisory Committee for Laboratory Animal Research and conducted following the guidelines approved by the Shanghai Ocean University Committee on the Use and Care of Animals.

### Family construction and breeding of goldfish

**Figure 7:**
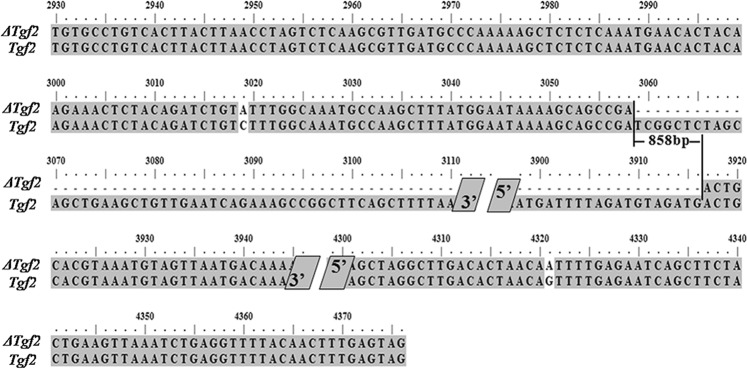
The sequence alignment of *Tgf2* and *ΔTgf2. Tgf2 was* the complete sequence type and *ΔTgf2* was the missing pieces type.

In breeding experiments, we observed that the twin-tail phenotype occurred in the wild-type family. From the mutation in the twin-tail goldfish genome, we identified two kinds of *Tgf2* transposon, one that was completely sequenced (*Tgf2*), and one with multiple pieces missing (*ΔTgf2*) (Fig. 7). To detect the relationship between the *Tgf2* transposon and the differentiation in the caudal fin, we constructed 10 families (*Tgf2* family-1–4 and *ΔTgf2* family-1–6) for analysis. Families were divided based on the results of *Tgf2* sequencing and each family contained 100 fishes. Primer set *Tgf2*-F1/-R1 (designed base on accession number HM146132.1, Table 3) was used to amplification *Tgf2*/*ΔTgf2* sequence.

**Table 3:** Primer sequences used in this study.

Primers name	Primer sequence (5′-3′)
chordinA-F1	TAACGCACAGATGCAGACGTGTG
chordinA -R1	TGCTGTTCTCCTCAGAGCTGATGTAGG
eve1-F	ATGCTCGCAGAGGGCAGGGAG
eve1-R	TCCTGAAGCACTGCCAAAGGTTTTGG
sizzled-F	ACGCTGCTCCACACCGGCTGTCACC
sizzled-R	GAACCGCTTCCTCCAGACTGCTGTGG
bmp4-F	CCTGGTAATCGAATGCCGATGGT
bmp4-R	GGCAGCCACATCCCTCCACC
krox20-F	ATGACAGCTAAAACTTTGGAG
krox20-R	GGGTTTGTGGCCGGTGTGATGC
Tgf2-F1	TGTGCCTGTCACTTACTTAACC
Tgf2-R1	CTACTCAAAGTTGTAAAACCTC
Tgf2-F2	CCATCATAAAACGAGGTAAA
Tgf2-R2	CTGCTTTAGAACTGTTTGTATTT

To obtain a genetically stable twin-tail goldfish strain, we crossed a wild-type goldfish (*ChordinA*^+/+^, ♂) with a mutant twin-tail goldfish (*ChordinA*^*ΔTgf2/ΔTgf2*^, ♀*)* to obtain the F1 hybrid *ChordinA*^+*/ΔTgf2*^. The F1 hybrid individuals were selfed to obtain F2 offspring. Twin-tail strains (*ChordinA*^*ΔTgf2/ΔTgf2*^) selected from the F2 were selfed to obtain F3 individuals. The pedigree structure is shown in Fig. 2a. The phenotypes of goldfish strains were based on the embryonic morphology at 2–3 dpf (days post fertilization) following previous studies^16,28^. All embryos were observed using a Nikon ECLIPSE 80i stereoscopic microscope (Tokyo, Japan). A total of 823 specimens were phenotyped at the embryonic stage.

### Anatomical and histological analyses of axial skeletons

For anatomical analysis, we referred to previous methods with appropriate improvements where necessary^37^. Juvenile goldfish specimens were fixed in 4% PFA solution and subsequently scaled and rinsed 3 times (for 30 min each time) using diethylpyrocarbonate (DEPC)-treated water. To eliminate the residual PFA stationary liquid and degrease, the specimens were rinsed twice (for 20 min each time) using Tris Buffered Saline Tween (TBST). In order to avoid pigmentation, the specimens were immersed in 1% KOH solution (potassium hydroxide with 1% H_2_O_2_) and exposed to strong high-lights. After being digested with trypsin, the specimens were rinsed and soaked with 1% KOH, then stained overnight with alizarin red solution (0.1% alizarin red in 95% ethanol). Finally, specimens were successively rinsed with mixed solutions (1% KOH:glycerinum at 3:1, 1:1, and 1:3).

For histological analysis, juvenile goldfish samples were fixed in Bouin’s solution. The specimens were dehydrated using a graded ethanol series, and made transparent using dimethylbenzene, embedded in paraffin, and then sliced into 3-μm sections using a slicer (RM2265, Leica). Finally, sections were stained using hematoxylin and eosin. All anatomical and histological samples were examined using a Nikon ECLIPSE 80i stereoscopic microscope (Tokyo, Japan).

### Probe preparation and sequencing

The probes of *ΔTgf2*, *Tgf2*, *chordinA*, *eve1*, *sizzled*, *bmp4* and *krox20* were prepared by using PCR. Total RNA was isolated from goldfish embryos at 44 hpf using TRIzol reagent (Invitrogen, Carlsbad, CA, USA) and subsequently treated with DNase (Promega, Madison, WI, USA) to eliminate contaminating genomic DNA. First-strand cDNA was reverse-transcribed from the total RNA using Reverse Transcriptase M-MLV (TaKaRa, Tokyo, Japan) with oligo-dT primers according to the manufacturer’s instructions. Primer sets *Tgf2*-F1/-R1, *Tgf2*-F2/-R2 (designed base on accession number HM146132.1), *chordinA*-F1/-R1 (designed base on accession number AB874473), *eve1*-F/-R, *sizzled*-F/-R, *bmp4*-F/-R and *krox20*-F/-R (refer to Gembu Abe) (Table 3) were used to amplification probes sequence^4^. PCR products were gel-purified, ligated into the T/A cloning vector pGEM-T (Promega, Madison, WI, USA) and transformed into *Escherichia coli* DH5α. Positive clones were examined by PCR and direct sequencing.

### Whole-mount *in situ* hybridization

Fixed goldfish embryos were washed briefly in PBS containing 0.1% Tween-20, then transferred to 100% methanol, and stored at −20 °C for a minimum of 24 h. Whole-mount *in situ* hybridization using digoxigenin (DIG)-labeled RNA riboprobes was performed as reported previously, with modifications^38^. Embryos were photographed using a Nikon SMZ1500 fluorescence microscope (Tokyo, Japan).
